# Clinicopathological Significance of NMIIA Overexpression in Human Gastric Cancer

**DOI:** 10.3390/ijms131115291

**Published:** 2012-11-19

**Authors:** Dongning Liu, Lei Zhang, Zhiyong Shen, Fei Tan, Yanfeng Hu, Jiang Yu, Guoxin Li

**Affiliations:** Department of General Surgery, Nanfang Hospital, Southern Medical University, Guangzhou, Guangdong 510515, China; E-Mails: liudongning198224@yahoo.com.cn (D.L.); sparkler1110@163.com (L.Z.); szy2728@163.com (Z.S.); isoktay_0@sohu.com (F.T.); yanfenghu1980@163.com (Y.H.); yujiang1974@126.com (J.Y.)

**Keywords:** gastric cancer, nonmuscle myosin IIA, survival

## Abstract

Altered expressions of nonmuscle myosin IIA (NMIIA) have been observed in certain types of cancers, but the impact of the alterations in gastric cancer (GC) remains unclear. The purpose of this study was to evaluate the expression of NMIIA at the mRNA and protein level in patients with GC and to assess its clinical significance. We investigated the expression of NMIIA in fresh, paired GC tissues by reverse transcriptase polymerase chain reaction (RT-PCR; *n* = 14) and Western blot analysis (*n* = 36). Simultaneously, we performed immunohistochemistry (IHC) on paraffin embedded specimens, including 96 GC specimens, 30 matched normal specimens and 30 paired metastatic lymph node samples. NMIIA is overexpressed in GC compared with the adjacent normal gastric epithelium (*p* < 0.001) and high-level NMIIA expression is significantly correlated with the depth of wall invasion, lymph node metastasis, distant metastasis and Tumor Node Metastasis (TNM) stage. Furthermore, elevated NMIIA expression is an independent prognostic factor in multivariate analysis using the Cox regression model (*p* = 0.021). These findings indicate that overexpression of NMIIA may contribute to the progression and poor prognosis of GC.

## 1. Introduction

Gastric cancer (GC) is a global health problem. There were an estimated 989,600 new cases and 738,000 cancer-related deaths in 2008 alone, with the highest incidence in Eastern Asia, Eastern Europe, and South America [1]. The molecular events involved in the development and progression of GC are complex, involving multiple genetic events that operate sequentially or in concert [2]. Several risk factors, including genetic alterations, chromosomal instability, and Helicobacter pylori infections, have been identified [3–5]. Currently, prognostic evaluation is based mainly on tumor site, clinical stage, and histopathologic grade. Recent studies have suggested that other factors, such as biological markers, may improve our ability to predict the prognosis and design treatment strategies [6,7].

Nonmuscle myosin IIA (NMIIA) is encoded by the MYH9 gene and belongs to the myosin II subfamily of actin-based molecular motors that includes skeletal, cardiac, smooth muscle, and nonmuscle myosins [8,9]. Although NMIIA reportedly participates in cell adhesion and migration, only a few studies have assessed its expression and role in cancer [10,11]. For example, epidermal growth factor-dependent phosphorylation of the NMIIA heavy chain directly mediates motility and chemotaxis in MDA-MB-231 human breast cancer cells [12]. In stage I primary lung adenocarcinoma, patients whose tumors lack NMIIA expression have significantly better outcomes regardless of postoperative adjuvant chemotherapy [13]. Another study of esophageal squamous cancer patients found a significant positive correlation between elevated NMIIA expression level and advanced tumor stage and lymph node metastasis. Further analysis suggested that strong NMIIA expression was associated with a significantly shorter overall survival [14].

It is thus interesting to test the expression of NMIIA and its role in GC. In this study, we investigated the expression of NMIIA by reverse transcriptase polymerase chain reaction (RT-PCR) and Western blot analysis in a large number of GC samples. We also performed immunohistochemical (IHC) analyses on a larger panel of GCs, matched normal gastric epithelial tissues and paired metastatic lymph nodes to determine whether NMIIA is upregulated in GC. Finally, we evaluated the relationship between NMIIA expression level and the clinicopathological parameters and prognosis of gastric carcinoma patients. To the best of our knowledge, this is the first report on evaluating expression of the NMIIA in clinical specimens of gastric carcinoma.

## 2. Results

### 2.1. Overexpression of NMIIA in Human GC

**Figure 1:**
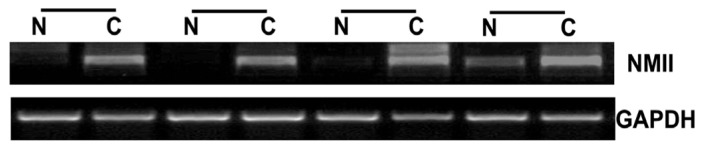
Overexpression of NMIIA in GC as detected by semiquantitative RT-PCR. Data presented here is a representative of all the samples. GAPDH was used as internal control. **N** indicates normal tissue; **C**, patient-matched tumor tissue.

To elucidate the role of NMIIA in the initiation and progression of GC, we first analyzed its expression in GC and matched adjacent normal tissues at the mRNA level. RT-PCR analysis of NMIIA expression in matched normal and tumor tissues showed that NMIIA was upregulated in the majority of GC tissues compared with their normal counterparts (71.50%, 10/14) (Figure 1).

### 2.2. Overexpression of NMIIA Protein in Human GC

**Figure 2:**
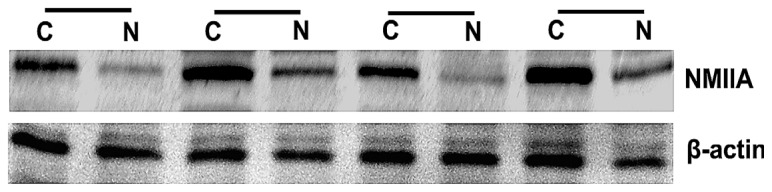
Overexpression of NMIIA in GC as detected by Western blot analysis. Data presented here is a representative of all the samples. β-actin was used as a loading control. **N** indicates normal tissue; **C**, patient-matched tumor tissue.

**Figure 3:**
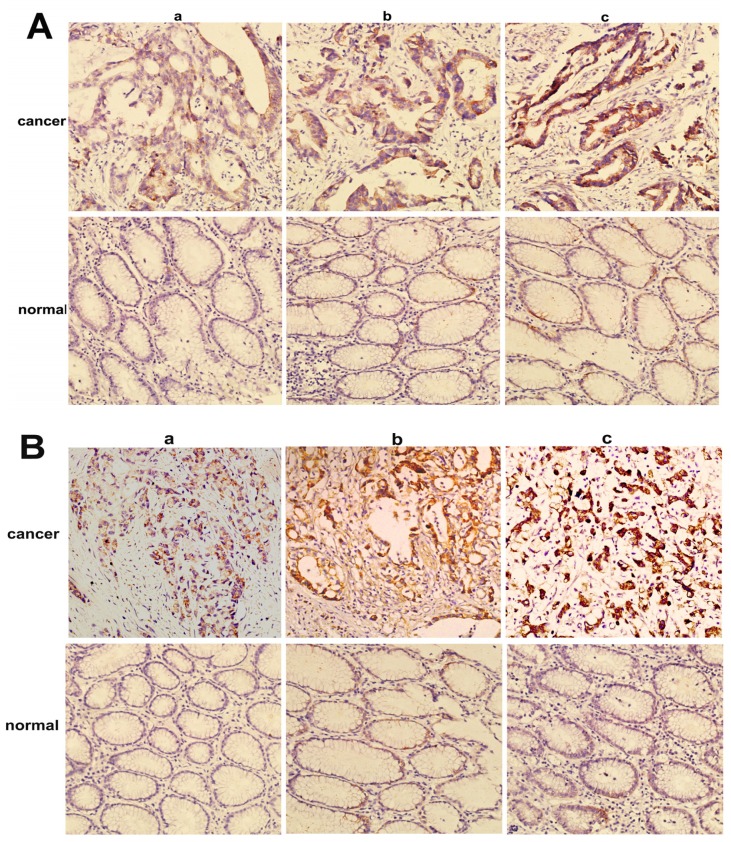
Immunohistochemical analysis of NMIIA expression in gastric carcinoma and adjacent normal gastric epithelium (200×). (**A**) Intestinal type gastric carcinoma; (**B**) Diffuse type gastric carcinoma. (**a**) Weak staining detected in tumor cells; (**b**) Moderate staining observed in tumor cells; (**c**) Strong staining detected in tumor cells.

**Figure 4:**
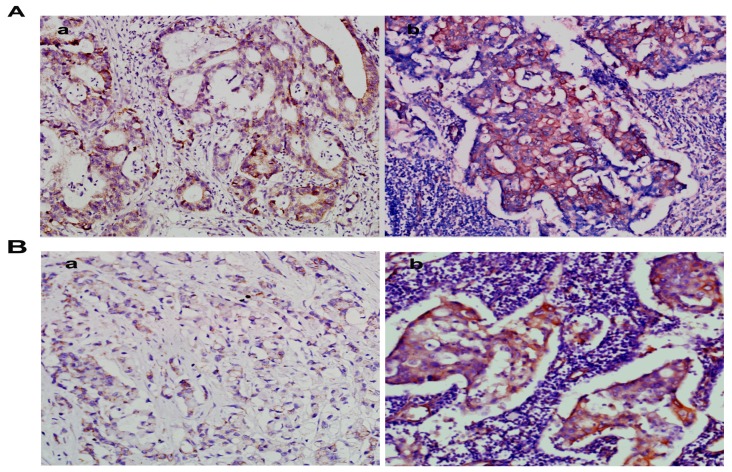
A gastric carcinoma sample shows weak NMIIA expression (**a**); but its metastatic lymph node has strong NMIIA expression (**b**) (200×). (**A**) Intestinal type gastric carcinoma; (**B**) Diffuse type gastric carcinoma.

NMIIA was similarly overexpressed at the protein level in GC as shown by Western blot analysis and IHC. The Western blot analysis showed that NMIIA protein levels were elevated in 26 of 36 (73.34%) GCs compared with normal tissues (Figure 2). Samples from the entire group of 96 patients that contained both cancerous and noncancerous tissues were evaluated for NMIIA protein expression by immunohistochemistry. Absent (6/30; 20.00%) or weak (24/30; 80.00%) NMIIA protein expression was detected in the mucosa of normal gastric tissues (Figure 3). In GCs, NMIIA was expressed in the cytoplasm of all cancer tissues and on the cell membrane of some cancer tissues. In 53 intestinal type gastric carcinomas, 28 (52.83%) showed strong NMIIA expression, 18 (33.96%) showed moderate NMIIA expression and 5 (13.21%) showed weak NMIIA expression (Figure 3A). In 43 diffuse type gastric carcinomas, 25 (58.13%) showed strong NMIIA expression, 12 (27.90%) showed moderate NMIIA expression and 8 (13.97%) showed weak NMIIA expression (Figure 3B). Low staining (SI < 10) was noted in 46 cases (47.91%), and high staining (SI ≥ 10) was shown in 50 cases (52.09%). The mean SIs of normal gastric tissue and GC tissue were 3.20 and 9.52, respectively. Compared with benign gastric tissue, NMIIA protein expression in GC is significant (16.92 *vs.* 78.06, *U* = 42.50, *p* < 0.001; Table 1). Furthermore, both in intestinal type GC (18.92 *vs.* 55.07, *U* = 102.50, *p* < 0.001; Table 1), and in diffuse type GC (15.57 *vs*. 51.95, *U* = 2.00, *p* < 0.001; Table 1), compared with benign gastric tissue, NMIIA protein expression in GC is also significant. All cancer cells in metastatic lymph nodes of 30 cases GC showed strong expression of NMIIA, whether their primary tumors were NMIIA high expression or low expression (Figure 4A,B).

### 2.3. NMIIA Overexpression in Relation to Clinicopathologic Parameters

Expression of NMIIA in tumor tissue was not significantly associated with age, gender, tumor location, tumor size, tumor differentiation, mean no. of tLNs, or mean no. of mLNs (Table 2). However, elevated NMIIA expression was strongly correlated with the depth of wall invasion (*p* = 0.026), mean no. of mLNs (*p* < 0.001), lymph node metastasis (*p* = 0.015), distant metastasis (*p* = 0.021) and TNM stage (*p* = 0.004) (Table 2).

### 2.4. Univariate Analysis

**Figure 5:**
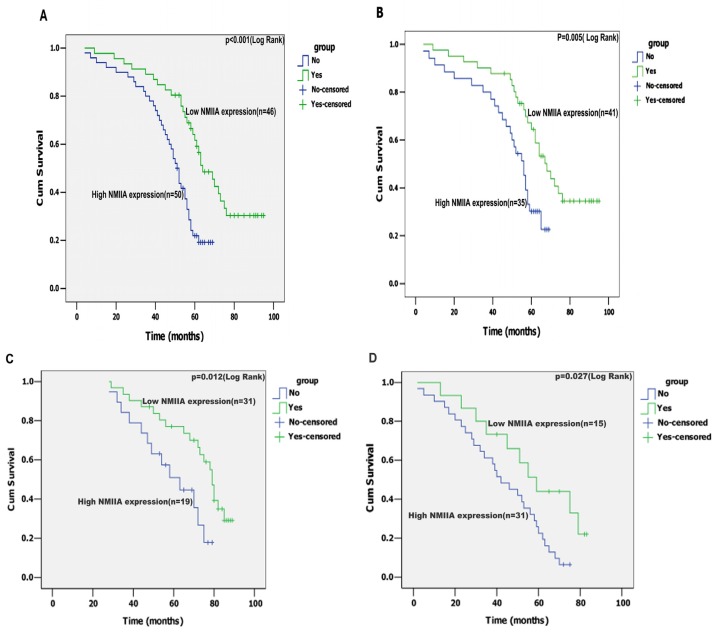

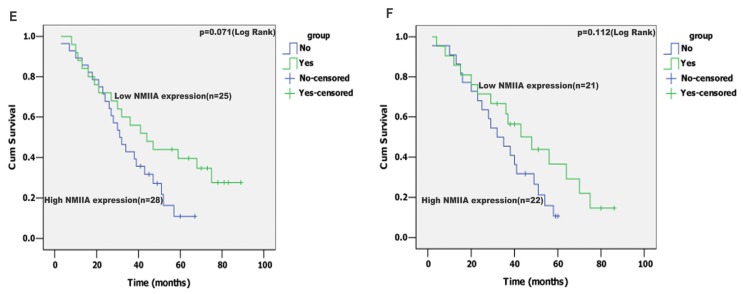
(**A**) The mean overall survival time for the high NMIIA expression group was 48.5 months, and for the low NMIIA expression group was 67.6 months (*p* < 0.001); (**B**) In R0 (radical operation) gastric cancer, the mean overall survival time for the high NMIIA expression group was 51.7 months and for the low NMIIA expression group was 69.8 months (*p* = 0.005); Statistical significance of the difference between curves of NMIIA high-expressing and low-expressing patients was compared within subgroups of TNM stage I + II (*p* = 0.012; **C**) and III + IV (*p* = 0.027; (**D**). No statistical significance of the difference between curves of NMIIA high-expressing and low-expressing patients was compared within subgroups of intestinal type gastric carcinoma (*p* = 0.071; **E**) and diffuse type gastric carcinoma (*p* = 0.112; **F**).

In univariate analysis, depth of wall invasion, lymph node metastasis, distant metastasis, TNM stage and NMIIA expression were associated with overall survival. A Kaplan-Meier analysis and the log-rank test showed that the survival time between the low and high level of NMIIA expression groups was significantly different (*n* = 96; *p* < 0.001, Figure 5A). Even in the curative resection cases, The difference in survival time was also significant (*n* = 76; *p* = 0.005, Figure 5B). Furthermore, the patients with tumors exhibiting high NMIIA expression had a significantly shorter overall survival time than those with low expression of NMIIA in either the TNM stage I + II subgroup (*n* = 50; *p* = 0.012; Figure 5C), or the TNM stage III + IV subgroup (*n* = 46; *p* = 0.027; Figure 5D). However, no significant correlation was detected between high NMIIA expression and shorter overall survival time within GCs of Lauren classification tested (intestinal type, or diffuse type), respectively (intestinal type, *p* = 0.071, *n* = 53, Figure 5E; diffuse type, *p* = 0.112, *n* =43, Figure 5F). These data suggest up-regulated NMIIA expression correlates with poor prognosis.

### 2.5. Multivariate Analysis

The following significant parameters were entered into a multivariate analysis: depth of wall invasion, lymph node metastasis, distant metastasis, TNM stage and NMIIA. The NMIIA expression was the independent predictor (HR = 2.031, *p* = 0.021; Table 3), which suggests NMIIA expression served as an independent prognostic factor.

## 3. Discussion

Gastric cancer (GC), the second most frequent cause of cancer-related death [1], remains a significant therapeutic challenge, and many molecular pathways implicated in its pathogenesis remain unknown. It is essential to understand the molecular mechanisms of tumor formation and progression to develop rational approaches to the diagnosis and treatment of cancer, and thus studies that seek to identify disregulated genes and proteins in neoplasms are critical. We thus focused on the newly characterized cancer-related gene, nonmuscle myosin IIA (NMIIA), which is involved in modulating tumor cell invasion and metastasis as well as predicting prognosis [12–18].

For this study, we investigated the NMIIA expression status in GC at both the mRNA and protein levels. The RT-PCR results revealed that of 14 paired cases, 10 showed higher NMIIA transcript levels in tumors than in normal tissues, and the Western blot analysis showed NMIIA protein overexpression in 26 of 36 GC tissues compared with matched normal tissue. Additionally, IHC of 96 paraffin-embedded GC tissue samples showed high expression of NMIIA protein compared with 30 normal epithelium samples (*p* < 0.001). The results showed that NMIIA was markedly overexpressed in human GC tissues compared with normal gastric epithelium. The mechanisms leading to NMIIA overexpression in human tumors are not well known. Possible mechanisms include point mutation, gene amplification, gene rearrangement and insertion of strong promoter or enhancer. Epigenetic modifications including demethylation and deacetylation may also be responsible. The mechanism of NMIIA overexpression in GC needs to be further investigated.

Cell migration involved cell protrusion, polarization, attachment of the leading edge to extracellular matrix, assembly (and ultimately disassembly) of adhesion plaques, and finally, retraction of the tail of the cell [11]. NMIIA reportedly participates in this series of steps [10,11]. NMIIA is also essential for the maturation of focal adhesion complexes in cell protrusions [19]. Ablation of NMIIA interfered with stress fiber formation, adhesion complex mature [20,21], and the disassembly of the adhesions at the cell rear [22], and thus impaired its retraction, thereby disrupting cell migration [22,23].

NMIIA has been implicated in a number of cellular activities that are directly or indirectly related to invasion and metastasis of malignant cancer [14,16–18]. For example, siRNA-mediated depletion of NMIIA inhibited esophageal squamous cancer cell migration [14]; this finding is consistent with a published study demonstrating impaired migration of NMIIA-depleted MDA-MB-231 breast cancer cells [16]. A significant positive correlation was also found between the expression levels of myosin light chain kinase (which activates NMIIA) and the likelihood of disease recurrence and metastasis for patients with non-small-cell lung carcinoma [17]. A large number of published studies have also focused on S100A4, a mediator of metastasis that is upregulated in GC and selectively binds to NMIIA [18]. In this study, increased NMIIA expression was observed in GC (both intestinal type GC and diffuse type GC) tissues, and all cancer cells in metastatic lymph nodes (*n* = 30) showed strong expression of NMIIA. Furthermore, an elevated NMIIA expression level correlated with the depth of wall invasion, mean no. of mLNs, lymph node metastasis, distant metastasis, and TNM stage. Collectively, these findings demonstrated that elevated NMIIA expression may promote GC cell invasion and metastasis.

Moreover, we analyzed the overall and curative resection (R0) patients’ survival rates in GC with low and high NMIIA expression, finding a statistical association between high NMIIA expression and poor prognosis. Furthermore, we found that the patients with high NMIIA expression had a worse outcome than those with low expression of NMIIA in either the TNM stage I + II subgroup, or the TNM stage III + IV subgroup. Nevertheless, no relationship between high NMIIA expression and shorter overall survival time was found in either the intestinal type subgroup, or the diffuse type subgroup. In addition, Cox proportional hazards models showed that high NMIIA expression maintained its independent prognostic impact on overall survival. NMIIA seems to be a strong predictor of poor prognosis for GC, in keeping with the findings in esophageal squamous cancer [14].

## 4. Materials and Methods

### 4.1. Tissue Specimens

Thirty-six fresh frozen GC and corresponding normal gastric mucosa tissue samples (more than 10 cm away from the edge of the GC) were taken from patients with GC within 30 min after resection, and then snap-frozen in liquid nitrogen and stored at −80 °C until use. Polyformaldehyde-fixed and paraffin-embedded GC tissue blocks (*n* = 96) were obtained from the stored files of the Department of General Surgery, Nangfang Hospital, Southern Medical University between January 2004 and December 2006. Additionally, 30 control samples from matched normal gastric tissues taken from the distal resection margin and 30 paired metastatic lymph nodes were collected. All patients had undergone preoperative clinical staging assessment with endoscopic ultrasonography (EUS) and multislice spiral CT (MSCT). No patients had received chemotherapy or radiotherapy before surgery. The various clinicopathological parameters (age, gender, tumor size, tumor location, tumor differentiation status, lauren classification, depth of wall invasion, lymph node metastasis and distant metastasis) were obtained from histopathology records. The details about surgical treatment performed are presented in Table S1. The stage of gastric cancer was described according to the 2010 tumor node metastasis (TNM) classification of malignant tumors by the American Joint Committee on Cancer (AJCC). Usage of GC specimens, matched normal specimens and paired metastatic lymph node samples for this study was approved by the Nangfang Hospital Ethical Committee.

The 96 patients included 59 males and 37 females aged 25 to 88 years (mean = 59.5 years). The patients were followed until death or the last follow-up date (30 November 2011). Complete follow up, ranging from 4 to 95 months, was available for all patients and the median patient survival was 57 months. Distant metastasis occurred in 20 cases (20.8%), including eight cases to the peritoneum, five cases to the liver, three cases to transverse colon, two cases to the pancreas and two cases to bone metastasis.

### 4.2. RNA Extraction and Reverse-Transcriptase PCR

Paired tissues were minced and total RNA was extracted with TRIZOL reagent (Invitrogen Life Technologies) and reverse-transcribed to first-strand cDNA with the TaqMan Reverse Transcription Kit (Applied Biosystems). Then, 0.5-μL to 1-μL aliquots of the cDNA were used as the template to amplify the *NMIIA* fragment (Forward primer: 5′-AGAGCTCACGTGCCTCAACG-3′; Reverse primer: 5′-TGACCACACAGAACAGGCCTG-3′) under the following conditions: 95 °C for 5 min; 28 cycles of 95 °C for 30 s, 57 °C for 30 s, and 72 °C for 30 s; and 72 °C for 5 min. The GAPDH gene was used as an internal control.

### 4.3. Protein Extraction and Western Blot Analysis

Western blots were performed according to standard methods as described by other authors [14]. A rabbit polyclonal NMIIA antibody (ab24762, Abcam, Cambridge, UK) was diluted 1:500 for Western blots. Tumor and adjacent normal tissues were frozen in liquid nitrogen and powdered with a mortar and pestle followed by lysis with cell lysis buffer. Samples were transferred to microcentrifuge tubes, homogenized, and protein pelleted by microcentrifugation at 14,000 rpm for 15 min at 4 °C. The protein concentrations were determined using the Bicinchoninic Acid Protein Assay Kit (Pierce, Rockford, IL, USA). The samples were separated electrophoretically on 10% SDS-polyacrylamide gels and transferred to a polyvinylidene difluoride membrane. After blocking, the membrane was incubated with the anti-NMIIA antibody at 4 °C overnight. After washing, the membranes were incubated with secondary antibody (Protein Simple, Santa Clara, CA, USA) at a dilution of 1:5000 at room temperature for 70 min. Proteins were detected with an enhanced chemiluminescence kit (Amersham Pharmacia Biotechnology Inc., Piscataway, NJ, USA), and an anti-β-actin antibody (mouse 1:1000 dilution, Sigma, Saint Louis, MO, USA) used as a loading control.

### 4.4. Immunohistochemistry

Immunohistochemistry (IHC) was performed as described by other authors [15]. In brief, the slides were dewaxed with xylene and rehydrated through an ethanol gradient into water. After endogenous peroxidase activity was quenched with 3% H_2_O_2_, sections were digested with 0.1% trypsin. After phosphate-buffered saline (PBS) washing, nonspecific antibody binding was blocked by incubating the slides with 10% normal goat nonimmune serum. Sections were incubated at 4 °C overnight with the rabbit polyclonal NMIIA antibody (ab24762, Abcam, Cambridge, UK) at a 1:200 dilution. After PBS washing, sections were incubated with a biotinylated secondary antibody and then with horseradish peroxidase-labeled streptavidin (Protein Simple, Santa Clara, CA, USA). After PBS washing, sections were developed using 3,3*V*-diaminobenzidine (Sigma, Saint Louis, MO, USA). Sections were washed in running tap water and lightly counterstained with hematoxylin, followed by dehydration and coverslip mounting. Negative control experiments were conducted by replacing the primary antibody with phosphate-buffered saline.

Immunostaining was scored semiquantitatively by two independent observers, Nan He and Liang Zhao, who were blinded to the patients’ outcome and other clinicopathologic parameters. Immunoreactivity was represented by an immunoscore in which the intensity of immunostaining was multiplied by its extent. There existed various degrees of background staining that may have been caused by fixation and embedding during tissue processing. Because such background staining is largely nonspecific and occurs in the stromal tissue, we avoided it by counting only positive epithelial cells. The edge effect was also regarded as negative. The NMIIA detection system has been described by other authors [16]. Based on the extent of immunoreactivity, each sample was assigned to one of the following categories: 0 = none; 1 ≤ 1%; 2 = 1%–10%; 3 = 10%–30%; 4 = 30%–70%; 5 ≥ 70%. The intensity of immunostaining was classified as: 0, negative; 1, weak; 2, moderate; or 3, strong. For each case, the immunostaining score, also known as the staining index (SI) was obtained as a measure of the intensity of NMIIA-positive staining and the proportion of immune-positive cells of interest were calculated. For this study, an optimal cut-off value was identified as follows: an SI score of 10 or higher corresponded to high NMIIA expression, whereas that between 0 and 9 denoted low/negative NMIIA expression.

### 4.5. Statistical Analysis

Statistical analysis was performed using the SPSS statistical software package (SPSS^®^ version 13.0, SPSS Inc., Chicago, IL, USA). Where appropriate, the Mann-Whitney *U*-test, 2-tailed *t* test, and Fisher’s exact test was used for comparisons between two independent groups, and the Pearson’s chi-square test was used for comparing more than two independent groups. Survival rates were calculated according to the Kaplan-Meier method and differences were evaluated using the log-rank test. Cox proportional hazards regression model was used to assess hazard ratio (HR) and identify factors that independently predict survival. Differences were considered significant if the *p*-value from a two-tailed test was < 0.05.

## 5. Conclusions

We have shown that expression of NMIIA may be used as a potential molecular marker to predict patient outcome in GC patients. Our data showed that high NMIIA expression has a high correlation with poor survival both in univariate and multivariate analyses. Therefore, future investigation on elucidation of the precise roles of NMIIA expression in GC will provide new insights, which will contribute to improved diagnosis and treatment.

## Figures and Tables

**Table 1:** Expression of NMIIA in benign *vs*. malignant gastric tissue. * Mann-Whitney *U*-test.

	*n*	SI (mean ± SE)	Mean rank	*U*	*p* * value
Benign epithelium	30	3.20 ± 0.26	16.92	42.50	<0.001
Gastric cancer	96	9.52 ± 0.29	78.06	-	-
Benign epithelium	30	3.20 ± 0.26	18.92	102.50	<0.001
Gastric cancer (intestinal)	53	8.91 ± 0.31	55.07	-	-
Benign epithelium	30	3.20 ± 0.26	15.57	2.00	<0.001
Gastric cancer (diffuse)	43	9.93 ± 0.24	51.95	-	-

NMIIA, Nonmuscle myosin IIA; SI, staining index; SE, standard error.

**Table 2:** Correlation between clinicopathological features and NMIIA expression.

Characteristics	NMIIA		*p* value

	Low (%)	High (%)	
**Gender**			1.000
Male	28 (47.45)	31 (52.55)	-
Female	18 (48.64)	19 (51.36)	-
**Age (years)**			1.000
<55	26 (47.27)	29 (52.73)	-
≥55	20 (48.78)	21 (51.22)	-
**Tumor size (cm)**			0.412
<5	23 (53.48)	20 (46.52)	-
≥5	23 (43.39)	30 (56.61)	-
**Tumor location**			0.548
Upper body or whole	21 (44.68)	26 (55.32)	-
Lower or middle body	25 (51.02)	24 (48.98)	-
**Tumor differentiation**			0.078
Well	20 (62.50)	12 (37.50)	-
Moderate	15 (46.87)	17 (53.13)	-
Poor	11 (34.37)	21 (65.63)	-
**Lauren classification**			1.000
Intestinal type	25 (47.16)	28 (52.84)	
Diffuse type	21 (48.83)	22 (51.17)	
**Depth of wall invasion**			0.026
T1	14 (70.00)	6 (30.00)	-
T2	12 (42.85)	9 (57.15)	-
T3	10 (47.61)	11 (52.39)	-
T4	10 (29.41)	24 (70.59)	-
**Mean no. of tLNs**	18	20	0.545
**Mean no. of mLNs**	7	13	<0.001
**Lymph node metastasis**			0.024
Absent (N0)	28 (60.86)	18 (39.14)	-
Present (N1–3)	18 (36.00)	32 (64.00)	-
**Distant metastasis**			0.025
Absent (M0)	41 (53.94)	35 (46.06)	-
Present (M1)	5 (25.00)	15 (75.00)	-
**TNM stage**	-	-	0.005
I + II	31 (62.00)	19 (38.00)	-
III + IV	15 (32.60)	31 (67.40)	-

NMIIA: Nonmuscle myosin IIA; tLNs indicates total removed Lymph Nodes; mLNs indicates metastatic Lymph Nodes; TNM: Tumor node metastasis; Distant metastasis included peritoneum, liver, transverse colon, pancreas, and bone metastasis.

**Table 3:** Results of univariate and multivariate survival analyses of overall survival by the Cox proportional hazards model.

Variable	Univariate			Multivariae		
		
	HR	CI (95%)	*p* value	HR	CI (95%)	*p* value
Gender	1.242	0.742–2.079	0.409	-	-	-
Age	0.997	0.973–1.022	0.815	-	-	-
Tumor size	1.007	0.995–1.020	0.266	-	-	-
Tumor location	1.176	0.721–1.917	0.516	-	-	-
Lauren classification	1.085	0.899–1.310	0.393	-	-	-
Tumor differentiation	1.063	0.792–1.426	0.684	-	-	-
Depth of wall invasion	1.818	1.431–2.309	<0.001	0.990	0.579–1.690	0.969
Lymph node metastasis	4.031	2.289–7.099	<0.001	2.849	1.388–5.850	0.004
Distant metastasis	4.069	2.262–7.320	<0.001	1.920	0.991–3.722	0.053
TNM stage	3.875	2.301–6.525	<0.001	2.474	0.432–5.028	0.535
NMIIA expression	2.530	1.485–4.311	0.001	2.031	1.113–3.706	0.021

HR indicates hazards ratio; 95% CI, 95% confidence interval; TNM, Tumor node metastasis; NMIIA, Nonmuscle myosin IIA.
